# Using online health information for unknown symptoms common among young adults: a qualitative analysis of health-related web pages illustrating the need for numeracy skills, the ability to deal with uncertainty, and the risk of ruling out self-care

**DOI:** 10.1080/02813432.2024.2408610

**Published:** 2024-09-27

**Authors:** Lisa Viktorsson, Eva Törnvall, Magnus Falk, Pia Yngman-Uhlin

**Affiliations:** a Research and Development Unit in Region Östergötland, and Department of Health, Medicine and Caring Sciences, Linköping University, Linköping, Sweden; b Management Department in Region Östergötland, and Department of Health, Medicine and Caring Sciences, Linköping University, Linköping, Sweden; c Primary Health Care Centre Kärna, and Department of Health, Medicine and Caring Sciences, Linköping University, Linköping, Sweden

**Keywords:** Online health information, young adult, health information website, healthcare guide service, health literacy, numeracy

## Abstract

Young adults experiencing unfamiliar symptoms commonly seek health information online. This study’s aim was to explore how health information websites express and communicate health information about symptoms common among young adults and guide readers in regard to health, illness, and care. Symptoms commonly searched for by young adults were used as search terms. The resulting data comprised material from 24 web pages and was analyzed using content analysis. The foremost purpose of online health information is to try to narrow down the user’s symptoms and then advise the user on what actions to take. This is done by first forming a foundation of knowledge through descriptions and explanations, then specifying the symptom’s time, duration, and location, and finally giving advice on whether to self-manage symptoms or seek additional information about them. However, the uncertainty of the diagnosis may rule out self-care. For readers inexperienced with health care, forming a decisive conclusion about diffuse symptoms on the sole basis of online health information could be challenging. The necessity of numeracy skills and the ability to deal with uncertainty are highlighted. There is a discrepancy between health advice given online and readers’ accessibility to health care that needs to be addressed in future policy and research.

## Introduction

Health information is frequently sought online by the general adult population, especially in regard to common symptoms with implications that are unclear to the individual seeking information [1,2]. In Sweden, over 90% of the adult population has daily access to the internet [3]. In combination with the fact that the Swedish health-care system struggles with having low perceived accessibility to primary health-care centers (PHCs), a preference for going online for answers to health questions or concerns is not surprising [4]. The possibilities of using the internet when searching for symptoms are numerous [2,5,6], but there are some important factors to consider before using online information [7]. Reviews of online information about specific diagnoses (endometriosis and periodontal diseases) have demonstrated that when factors such as the quality, accuracy, and credibility of online information are considered, the selection of reliable sources is low [8,9]. In addition, research has shown that only one-third of patients who visit health-care facilities receive the diagnosis suggested by their earlier online search [10]. Emergency department patients with non-urgent symptoms have been shown to use more online information than do PHC patients [10,11]. In the long term, this raises the question of whether online health information is contributing to potentially avoidable healthcare visits.

Health literacy is important to consider when studying online health information, primarily as it affects users’ understanding of health information, but also it has been shown to be associated with health-care utilization [12–14]. Health literacy is defined as the capacity to access, understand, appraise, and apply different types of health information in order to make decisions about health [15]. It comprises a range of knowledge skills regarding health information, from basic reading comprehension and content assimilation to critical analysis [16]. In addition, there is also e-health literacy specifically concerning the capacities to engage in own health and digital services, feeling safe and in control, and having access to systems that work and suit individual needs [17]. An earlier study demonstrated that online health information primarily benefits the already health literate [18], and another has recommended that material intended to inform patients be written at a sixth-grade reading level [19].

Young adults (18–29 years) have in several studies been shown to be one class of contributor to avoidable health-care visits [20,21]. Younger age is a predictor of frequent use of internet information [22], but younger age also means inexperience with adult health care [23]. This age group has been shown to be insecure about handling symptoms with self-care, preferring instead to seek professional health care for even minor illnesses [24]. The ability to define bodily sensations and give them meaning; the intensity, quality, and duration, together with the degree of associated stress, can be difficult since it involves several levels of interpretation skills [25]. Health-care providers have supported the above statements by presenting their experience of young adults having difficulties separating normal bodily changes from symptoms in need of health care [26]. Young adults may lack the experience, knowledge, and/or confidence needed to handle and interpret both their symptoms and information about symptoms. Based on the considerations above, there is interest in examining the existing online health information about ill-defined symptoms common in younger adults.

## Aim

The aim of the study was to explore how health information websites express and communicate health information for symptoms common in young adults and guide readers in regard to health, illness, and care.

## Method

The study has an explorative qualitative design that uses content analysis according to Patton [27].

### Study population and data collection

#### Setting

In Sweden, health care is publicly funded. The majority of health-care visits occur at PHCs, preferably after the patient contacts the national health-care guide service platform (named for its phone number, 1177) by telephone or online. After an initial visit at the PHC, a referral to further specialist health care is issued if considered needed. If symptoms are urgent or an injury is severe, the patient is expected to visit an emergency department or book an appointment at an on-call center, especially when PHCs are closed (weekends and after working hours). Although health care is publicly funded, private alternatives for outpatient care have increased in Sweden in recent decades as a result of political reform, namely the Act on the System of Choice in the Public Sector [28]. The Act recognizes every individual’s right to choose their provider and has enabled the establishment of more private health-care companies than before [29]. In addition, the Patient Act was introduced in 2014 with the objective of strengthening patients’ ability to influence their own care by, for example, enabling patients’ access to their medical records online [30].

#### Data collection

Data collection from online sources started in February 2020. The Swedish country code top-level domain (.se) was used and google.se was chosen as the search engine. Other search engines, Bing and Start page, were tested and gave results similar to those from Google. The choice of search terms was based on commonly researched symptoms among young adults seeking health care. Three symptoms were identified from previous research [31]: stomachache, chest pain, and headache. The search terms generated millions of websites, which is why the search results were limited to include only the first page of results. This decision was based on research showing that internet users seldom continue to the second page of results [32,33]. An initial overview of the top 50 results for each search term confirmed the validity of this exclusion. The included search results comprised commonly used Swedish websites containing information regarding health, illness, and health care.

**Figure 1.**
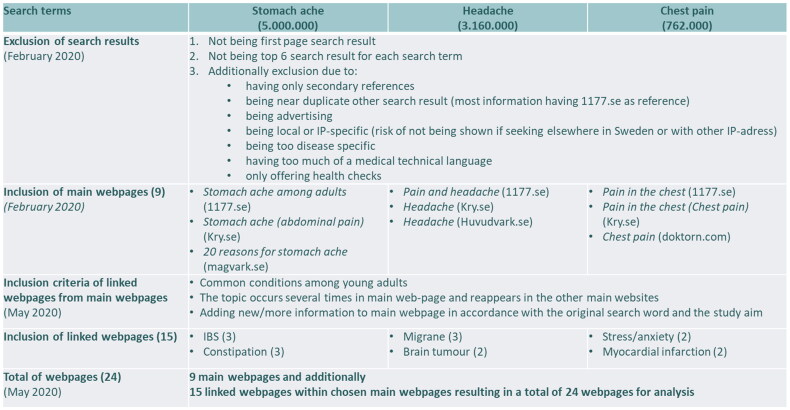
Flow chart of data collection. Numbers of websites and web pages given in parentheses.

The second step of data collection was done with purposeful sampling. In the collected websites, the chosen search terms generated broad health information with multiple opportunities to read additional information by clicking on links. Since websites are not built up in a linear manner like books, linked web pages were viewed as well. Linked web pages later became a second part of the data collection, in May 2020. To be included, they had to meet the following three criteria: providing information on a common condition in young adults, presenting a topic reappearing on the main website as well as on the other main websites, and having to add new/more information not already presented on the main website while still being an internal web page. All criteria for inclusion were defined based on findings in previously conducted studies [23,26,31]. The inclusion of linked web pages necessitated narrowing our analysis from the initial whole first page of results to make a cut off after the top 6 results. This excluded 8 websites from the collected data. This was done both to keep the amount of data manageable and to focus on the data most suited to the aim of the study, and yielded 18 websites. Of these, 9 were excluded foremost because they were secondary references, had too much technical language, were too specific within the searched area, or were near duplicates. The included websites had links to nearly 100 additional web pages. Of those web pages, 15 were chosen for inclusion: 6 pages about stomach ache, 5 about headache, and 4 about chest pain. These 9 websites and the additional 15 website pages linked within those websites provided 24 online health-information texts, which ranged from 540 to 3400 words. A detailed description of the inclusion process is presented in Figure 1. Included texts were copied and pasted into Word documents and then transferred into NVivo to facilitate text analysis.

### Analysis

Texts were analyzed using content analysis according to Patton [27]. First, the texts were read through and field notes and comments were written in a first attempt to organize the text into topics. In the second step, all texts were read through once again and systematically coded. When developing codes and, later on, categories, we continually searched for patterns. This included looking for recurring regularities, data that belonged together (internal homogeneity), and data that clearly differed (external heterogeneity). In the third step, we searched for categories by going deeper into the data and looking for connections in the data (and testing new categories). The initial categories showed a pattern with dimensions suitable for transfer to what Patton calls a logical analysis [27]. The dimensions of categories were cross-classified to try to generate new insights into the data and enable a deeper understanding of the patterns. This also became an attempt to test the initial coding and category setting for consistency. With the use of a logical construct, and going back and forth between categories and data, a more well-defined and simplified matrix emerged that fitted the data. Throughout the analysis, health literacy was considered but not explicitly measured. The first author, PhD student and public health scientist (LV), conducted all text analysis with the assistance of authors ET and PYU, all having previous experience in qualitative analysis. All three authors read and compared codes and categories with original texts in search of consistency.

**Figure 2.**
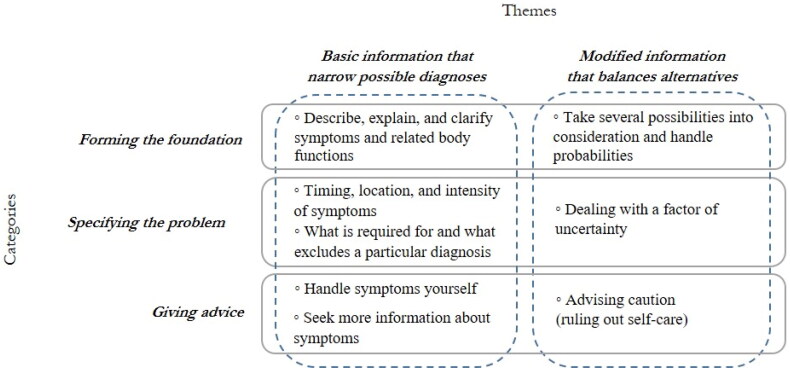
Analysis of health information online: Balancing elemental with modified information.

## Results

The results show that health information websites first and foremost provide and express basic health information by narrowing down possible diagnoses through three main categories: *forming the foundation, specifying the problem*, and *giving advice* (Figure 2). However, within the same three categories, the information also emerges as modified, with the reader having to balance alternatives, allowing for multiple possible diagnoses. This is illustrated by two themes. The first theme, *Basic information that narrows possible diagnoses* comprises basic information with a focus on identifying symptoms and giving suggestions for handling particular symptoms. This part is core health information: giving a message in a concrete form, mostly by trying to target the possible underlying diagnosis and giving solutions. The second theme, *Modified information that balances alternatives* emphasizes a more complex dimension of information. There are multiple, sometimes aggravating factors added to the basic information that require more from the reader in terms of knowledge and interpretive skills. All citations in the results are coded with a letter representing a specific web page.

### Forming the foundation

The information that forms the foundation *describes, explains, and clarifies symptoms and related body functions* by using examples, giving suggestions, and making comparisons, the information aids the reader in identifying symptoms and their cause. Much of the information is about trying to describe the type of pain that is normally and/or usually associated with a certain condition. This category also contains information focused on clarifying and explaining the language used. There is also clarifying information on why the body acts the way it does and why symptoms occur. Recurring throughout the texts are different types of words commonly used in health-care settings. When the information is basic, these words are defined and their relevance is explained.

If you have kidney stones you will most likely be in so much pain that you will find it difficult to lie still and find a pain-free position. (A)

Various factors can trigger a migraine. Common triggers are hormonal changes, certain foods – such as chocolate, strong cheese and red wine – strong odors, lack of sleep and changes in the weather.(T)

Forming the foundation appears elemental, but there is also a modified dimension where information concerns having to *take several possibilities into consideration and handle probabilities*. For instance, the reader has to distinguish between many different kinds of symptoms, whether they are harmful or harmless, and if the symptom they have is a sign of sickness or just a normal bodily sensation. The reader also has to consider that symptoms differ between people and can vary over time and between different individuals. Concepts are expressed in general terms. Symptoms are described as common or unusual, and risks are discussed, sometimes with statistics and by making generalizations. Probabilities are presented, but without explanations of how they should be interpreted.

There can be many causes of chest pain, varying from temporary and benign ones, to serious conditions that require acute treatment. (D)

‘More than 50 percent of acute chest pain is related to something other than the heart. (D)

### Specifying the problem

One way to help the reader identify their condition is to map the symptoms to *timing, location, and intensity*. Where on the body does it hurt, for how long has the ache been going on, and what is the intensity of the ache are three recurrent questions. The information in this category is to a great extent time related, regarding when the symptoms started, how the body behaved before the symptoms started, whether there have been any differences in symptoms during their duration, and whether symptoms can be classified as temporary or long lasting.

The pain is often felt on the left side of the abdomen, radiating to the back. (V)

Pre-conditions, that is, *what is required for and what excludes a particular diagnosis,* are used to help identify and differentiate symptoms. Some pre-conditions, such as age and sex, are easy to determine. Whereas time-related pre-conditions requiring the reader to recall, for example, what happened in connection to the appearance of symptoms may be more difficult to determine.

To receive a diagnosis of IBS [irritable bowel syndrome] you will have abdominal pain combined with one or more of the following symptoms… (O)

Information aimed at diagnosing the conditions associated with different symptoms also includes a modified dimension of trying to *deal with a factor of uncertainty*. This is expressed in many ways throughout the texts with expressions like ‘being mistaken for’, ‘being difficult to determine’, ‘being dependent on many different things’, ‘may be X but could also be Y’, etc. The information is written so as to try to take into account every possible aspect of having a symptom. The information is also ambiguous and hedged, focusing on declaring exceptions. Health information in this category is characterized by veering between ‘nothing to be worried about’ and ‘unusual but serious’: symptoms are explained either as almost never indicating something serious, or as indicating serious conditions a person may have without knowing it. Texts can calm or provoke worry, and sometimes they do both in the same sentence.

Chest pain may be caused by everything from indigestion, compression syndrome and stress, to pulmonary disease and serious cardiovascular problems. (D)

Intense and sudden headaches in connection with exertion may indicate that you have had a cerebral hemorrhage, but this is very rare. (L)

Constipation is not usually dangerous, but may sometimes be a symptom of a disease. (E)

### Giving advice

*Handle symptoms yourself* consist of the advice to wait, cope, or exercise self-care. The advice to wait is based on having narrowed the possible conditions down to ones with symptoms that most likely will go away with time. When information is given to the effect that symptoms are probably nothing serious and will go away, this is done in either a passive or a supportive way. The advice to handle symptoms by simply waiting is primarily communicated in a supportive way, meaning information is followed up with suggestions and encouragement aimed at easing worries and pain or just calming the reader. Information encourages the reader to recognize, understand, and learn about conditions and symptoms, thereby enabling self-care. When the information is communicated in a passive way, the information only tells you to wait without further explanation, suggestions, or encouragement. Lastly, there are recurring suggestions in the information about taking actions to prevent symptoms from occurring or recurring, although some of the actions suggested impossible to achieve if already having symptoms.

Everyone experiences stress sometimes – some people more than others. Different things cause stress for different people, but it is possible to learn to manage stress. If you are experiencing stress it is important to give yourself time to recover. (X)

Long-term anxiety can make you more sensitive to stress; you may feel restless or impatient, or become easily irritated. You may also have difficulty sleeping. If you use alcohol or drugs to ease your anxiety there is a risk of developing an addiction. It is therefore important to learn to manage your anxiety in other ways. (Y)

There are three main ways the reader is encouraged to *seek more information about their symptoms*: ask or consult people who are more knowledgeable, read more on other web pages, or go to a health-care facility. When referring to people who are more knowledgeable, the websites mostly mention facilities rather than a specific person: these include pharmacies, primary health-care centers, and emergency departments. The websites mention contacting specific professionals such as psychotherapists, doctors, and physiotherapists. This is sometimes expressed in terms of asking *your* doctor, thus assuming that the reader already has a doctor.

When a website encourages the reader to seek more information about their symptoms, it is based on the reader’s own suspicions and/or fulfilled conditions. The reader is encouraged to ask others for more information or consult a health-care professional. In this aspect, information provided by private health-care companies differs somewhat from that provided by the public health-care system. Information managed by private health information companies explains what they can offer the patient. Information provided by the public health-care system focus on patients’ rights according to principles of patient-centered care, including patients’ right to be involved in their own care.

If you suspect IBS you should see a doctor to rule out another diagnosis. (A)

You should, for example, get information about treatment options and how long you may need to wait to receive care and treatment. (L)

Get the right help wherever you are within just a few minutes.

- *A doctor’s appointment and session with a psychotherapist via video.*
- *Prescriptions and referrals as needed.*
- *Open 24 hours a day, every day of the year.’* (D)

*Giving advice* also adds a dimension of modified information by sometimes offering solutions or helping with such *caution* that it might implicitly rule out self-care. The need for diagnosis, knowledge from a certain medical profession, particular medical treatment, or investigation is highlighted, communicating the need for a health-care visit. The information provided covers various treatments and medical investigations needed to handle the reader’s symptoms.

Since there are many different causes of abdominal pain, the treatment required will vary greatly depending on what is causing it. If you can identify where exactly you feel pain – on one side, in the upper or lower abdomen – this will provide important information for diagnosis. (U)

Abdominal pain may be a sign of many different things because the abdomen is the area of the body where most of the organs are found. Abdominal pain should therefore always be taken seriously before a doctor has had a chance to make a diagnosis. (A)

There are many different causes of chest pain. It is therefore best to be examined by a doctor if you are unsure of what is causing it. (B)

## Discussion

As illustrated by this study, the online health information we explored is primarily basic in its content, consisting of well-structured sections aimed at helping the reader decide what to do about their symptoms. However, the information also contains a modified, more complex dimension with material that omits needed details, opening it up for interpretation, questions, and/or uncertainty. The modified dimension, to be considered the study’s main finding, demonstrates the need to consider several possibilities and to manage probabilities. Reading online health information means dealing with factors of uncertainty and being advised caution, in some cases ruling out the possibility of self-care.

Having basic and easily understood health information is probably the goal of most online health information services. However, having a modified dimension as part of the information is probably inevitable, since it is not possible to easily determine, on a generalized level, the cause of every symptom. These study results, demonstrating that online health information oscillates between basic and modified, influencing a reader’s potential for self-care, correspond well with findings from other research [34]. Factors often mentioned in favor of online health information, such as privacy and convenience [5,6], could become barriers to understanding and/or decision making, since the internet offers a large quantity of information that the reader might have difficulty interpreting and analyzing. In a face-to-face health-care visit, it can be argued that the modified dimension of the information is in the hands of the health-care professional to analyze and sort out for the patient. With online information, the reader has to interpret the information by himself or herself, managing probabilities and uncertainty in order to balance risks and decide if it is safe to handle their symptoms with self-care. This study adds an important argument in favor of the need for encounters between patients and health care professionals, not having health care professionals being replaced by online health information. In addition, the study further demonstrates the need for sufficient health literacy to fully grasp information online.

Considering readers’ health literacy when developing online health information is arguably essential. According to research, it is recommended that health information use a sixth-grade language level to be understood by the general populace [19]. The main findings from this study – the health information having a modified dimension repeatedly referring to risks and probabilities – support the need for online health information to consider readers’ numeracy skills as well; their ability to understand and use numbers in daily life. Young adults in Sweden have shown to be active consumers of online health information. They also rate health information online as good and trust it, though they also acknowledge that its quality varies, depending on the source [35]. Young people often show confidence in knowing how to use and find health information online. However, patients also demonstrate difficulty interpreting risks and probabilities [36], and recipients with poor numeracy skills are less accurate in their perceptions of risk levels [37,38]. When developing online health information, taking both health literacy and numeracy into consideration could possibly improve the receiver’s comprehension of the information [36]. Ultimately, online health information could benefit from being analyzed in regard to both health literacy and numeracy to address the needs of the public and reduce the risk of misinterpretation.

The study results demonstrate that online health information gives readers two options: to handle symptoms themselves or seek further help, two alternatives probably perceived as straightforward and relatively easy to clarify. However, by advising caution, the readers are encouraged to consult and visit professionals, with the risk of reducing the reader’s confidence in practicing self-care. The modified dimension of information repeatedly asserts the need for diagnosis or assessment by a healthcare professional to be certain of the symptom’s cause, thereby ruling out self-care. Self-care, which is possibly already challenging for many, could become even more challenging if the reader is given information on the advantages of professional assessment and diagnosis. The results demonstrate a gap between the information online and information that can be obtained in health-care settings. The suggestions on where to go and who to consult occasionally poorly reflect reality. For instance, the advice to ‘ask your doctor’ could be perceived as problematic, since, in Sweden, few patients can easily make direct contact with a doctor. An earlier study about health-care personnel’s perceptions of young adults’ health-care seeking behaviors showed that young adults are often perceived as transferring too much responsibility for their own health to the health-care system [26]. Health-care personnel also perceived young adults as sometimes seeking care from the wrong health-care setting. Examples given were young adults seeking emergent care and demanding to see a doctor for minor symptoms [26]. Online health information could be a possible source of this change in perceived responsibility.

### Discussion of method

In analyzing texts, it is difficult to separate qualitative and quantitative analysis completely, as both are needed: it is important to comprehend the underlying meaning of certain words and sentences [39], along with the frequency with which content appears. That is why this study, though having a qualitative approach, has also considered the frequencies of words and meanings sometimes being expressed in the results. A decisive part of text analysis is the merging of texts with the contexts of contemporary society; in this study, this means the social acceptance of consuming health-care information from different sources. The online health information explored in this study briefly highlights a contemporary society of trying to initiate transition from traditional health care setting with a hierarchical (patriarchal) receiving of care to more co-production. Earlier studies have demonstrated this transition as well and have been of influence on the analytical process of this study [23,26,31].

The study has limitations. For example, Google was chosen as the only search engine for data collection. However, the research team did try two other search engines, which gave similar results. This study’s aim was not to deliver a structured review of online health information in general, but to explore how online health information can be perceived from the readers’ point of view, with a specific age group in mind. One challenge in this study was the uncertainty of the chosen websites; the authors could not know if chosen websites were in fact the most frequently consulted websites among young adults. The authors handled this by scrutinizing all search results on the first result page together with the use of inclusion and exclusion criteria based on earlier research, both from the authors themselves and from others [23,26,31–33]. A possible limitation concerning transferability was the decision to make a cut-off after the first six top results, with the result that kry.se and 1177.se appear overrepresented. At the time when the data was collected, kry.se and 1177.se were the two most represented web pages irrespective of the search word, and had most information about the different symptoms. In addition, the choice of 1177.se and kry.se was the result of purposeful sampling, having the need for both publicly funded health care (1177.se) and private health care (kry.se) websites. Another possible limitation that needed to be addressed was the selection of linked web pages. When collecting the data, it was important only to choose texts relevant to young adult consumers. Again, by using purposeful sampling, the authors used knowledge and experience about young adults’ health care utilization from previously conducted studies as an starting point for what was to be included concerning linked web pages [23,26,31].

## Conclusion

This study demonstrates multiple challenges in coming to a decisive conclusion about diffuse symptoms using online health information as the only resource when inexperienced with health care. The necessity of numeracy skills and the ability to deal with uncertainty are highlighted. There is a discrepancy between health advice given online and reader’s accessibility to health care that needs to be addressed in future policy and research.

## Supplementary Material

Appendix categorization 240913.docx
