# Supplementary material for: Using online health information for unknown symptoms common among young adults: a qualitative analysis of health-related web pages illustrating the need for numeracy skills, the ability to deal with uncertainty, and the risk of ruling out self-care
Source: Scand J Prim Health Care. 2024 Sep 27;43(1):131–9. doi: 10.1080/02813432.2024.2408610 (PMC11834765; doi:10.1080/02813432.2024.2408610)
Supplement: Appendix categorization 240913.docx [file IPRI_A_2408610_SM5469.docx]

| **Main categories** | **Categories** | **Sub-categories** | **Examples of coding** |
| --- | --- | --- | --- |
| Forming the foundation | Describe, explain and clarify symptoms and related body functions | Explaining the diagnosis | - Constipation is the result off fiber and liquid… |
|  |  | Defining medical terms and clarifying definitions | - In medical terms called… |
|  |  | Explaining the symptoms | - Appears very suddenly and rapidly becomes more intense |
|  |  | Explaining treatment, examining and medical investigation | - Example of treatment other than drugs for headache |
|  |  | Clarifying how the body works | - Explaining why our body reacts with anxiety |
|  | Take several possibilities into consideration and handle probabilities | Distinguishing between different kinds of symptoms | - You may be scared of having pain, making you anxious, which may worsen the pain - The symptoms may vary from harmless to severe in need of emergency care |
|  |  | Setting in relation to other factors | - The pain is stronger, more persistent, and does not go away, even after resting |
|  |  |  | - The symptoms differ between different people |
|  |  | Common or unusual, and whether there is a risk of having or a chance of not having a condition | - Normally, the symptoms will disappear in a couple of days - Untreated can be life-threatening - 1,300 people suffer from a brain tumor each year |
| Specifying the problem | Timing, location, and intensity of symptoms | Locate where in the body | - Sore in case of contact with upper abdomen - Sore when putting pressure on sternum |
|  |  | Explaining grades of pain | - Causes so much pain that you become pale and puts you in a cold sweat |
|  |  | Time-related symptoms | - Recurring pain over a long period of time - A short period before you get ill you will have… - Manifests in very sudden, intense pain |
|  | What is required for and what excludes a particular diagnosis | Pre-conditions | - In connection with eating something - The pain often occurs in connection with physical exercise and stress - Could be a case of… if you at the same time… |
|  |  | Giving examples and/or suggestions based on certain criteria | - Certain pharmaceuticals can be the cause of constipation |
|  | Dealing with a factor of uncertainty | Can be caused by many different things | - Can be caused by many different things, from harmless to severe - Many different reasons for chest pain |
|  |  | Negative associations | - Rare but may be caused by severe illness - Mostly harmless but could be a sign of… |
|  |  | Difficult to decide or determine | - Symptoms can easily be mistaken for something else - Difficult to determine whether symptoms are hazardous or harmless |
| Giving advice | Handle symptoms yourself | Wait, it will pass, or wait to see what happens | - Symptoms are nothing to worry about and will go away by themselves |
|  |  | How to cope with and/or ease symptoms | - Learn how to reduce experiences of stress - Give examples of how to cope with anxiety |
|  |  | Handle with self-care | - Symptoms usually go away if you… - Reduce social associations affecting you negatively |
|  | Seek more information about symptoms | Consult expert | - Ask your doctor for advice - Ask a doctor or pharmacist |
|  |  | Circumstances when to seek different kinds of care | - Circumstances when to seek emergency care - Contact healthcare if you are having recurring periods of pain over a long period of time - If you have suspicions or are unsure, seek care |
|  |  | References to where and how to find out more | - You can find out more here… |
|  |  | Help is available | - We can help you with ailments - Best to let a doctor decide - Possible to get good treatment |
|  | Advising caution (ruling out self-care) | Requiring management from healthcare | - You should contact healthcare if… - To be able to diagnose you must… - This will be prescribed to you by your doctor |
|  |  | Treatment requiring diagnosis | - If you have … there are medications that can help - There are different treatments depending on illness |
